# From Mo–Si–B to Mo–Ti–Si–B Alloys: A Short Review

**DOI:** 10.3390/ma16010003

**Published:** 2022-12-20

**Authors:** Mi Zhao, Wei Ye, Mengyuan Zhu, Yuteng Gui, Wei Guo, Shusen Wu, Youwei Yan

**Affiliations:** 1 School of Aerospace Engineering, Huazhong University of Science and Technology, 1037 Luoyu Road, Wuhan 430074, China; 2 State Key Lab of Materials Processing and Die & Mould Technology, School of Materials Science and Engineering, Huazhong University of Science and Technology, 1037 Luoyu Road, Wuhan 430074, China; 3 Research Institute of Huazhong University of Science and Technology in Shenzhen, Shenzhen 518057, China; 4 State Key Laboratory for Mechanical Behavior of Materials, Xi’an Jiaotong University, Xi’an 710049, China

**Keywords:** Mo–Ti–Si–B alloys, alloy design, microstructure, mechanical properties, oxidation resistance

## Abstract

Mo–Si–B alloys have attracted considerable research interest during the last several decades due to their high melting points, excellent high-temperature strength and relatively good oxidation resistance. However, insufficient room-temperature fracture toughness and high-temperature oxidation resistance restrain their further application. Generally, a sufficient volume fraction of BCC-Mo solid-solution phase, providing the ductility, and a high Si content, responsible for the formation of passive oxide scales, is difficult to achieve simultaneously in this ternary system. Recently, macroalloying of Ti has been proposed to establish a novel phase equilibrium with a combination of enough BCC phase and intermetallic compounds that contain a large amount of Si. In this article, the development history from the ternary Mo–Si–B to the quaternary Mo–Ti–Si–B system was reviewed. It was found that the constitution phases could be easily tailored by changing the Ti content. In this regard, better performance of mechanical properties and oxidation resistance can be obtained through proper alloy design. In-depth understanding of the advantages of the quaternary alloys over their ternary ancestors may contribute to bringing about a new concept in designing novel ultra-high-temperature structural materials.

## 1. Introduction

Elevating the operating temperatures of aircraft engines and land-based power plants is one of the greatest challenges for the rapid development of modern industry. For a long period, the progress in high-temperature technology has largely relied on Ni-based superalloys that possess an amazing combination of toughness, high-temperature creep resistance and corrosion resistance [1,2,3]. In the ultra-high-temperature range where jet engines work, turbine blades have to be protected by thermal barrier coatings (TBCs) [4] and cooling systems due to the limited melting point of Ni-based superalloys. In this case, efficiency losses inevitably generated are attributed to the cooling approaches [1]. Therefore, the development of new structural materials is no doubt essential to solve the trade-off between increased operating temperature and efficiency losses in advanced jet turbine engines.

**Figure 1:**
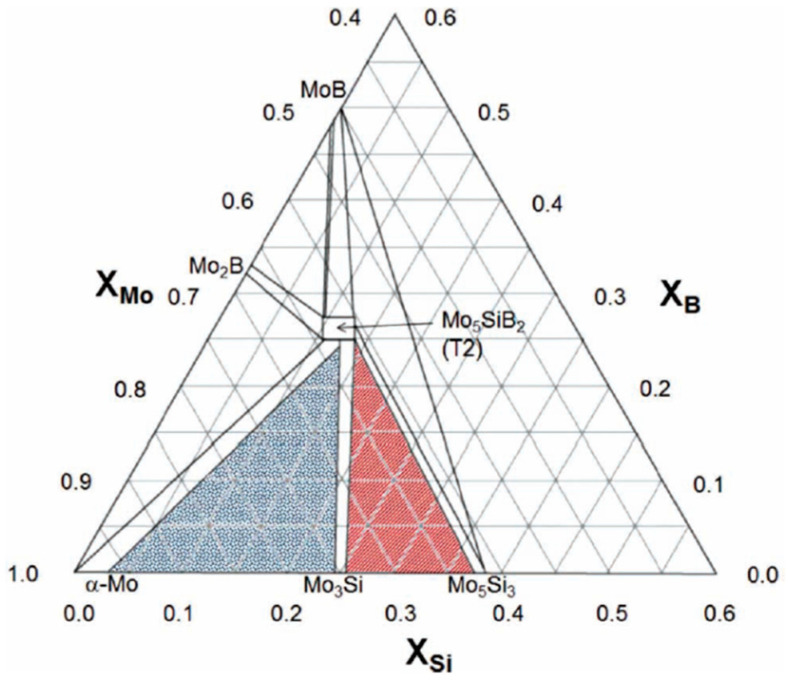
Diagram showing the Mo-rich portion of the 1600 °C isotherm of the Mo–Si–B phase [16].

Refractory metals and their silicides, mainly based on Nb and Mo, are promising candidates for the next generation of ultra-high-temperature applications, benefiting from their high melting points [5,6,7,8,9]. For example, in situ Mo–Si–B composites have attracted considerable scientific research interest since the 1990s due to their high thermal stability and excellent high-temperature creep resistance [10,11,12,13,14,15]. The most commonly studied Mo–Si–B system is composed of BCC-Mo-rich solid solution (BCC-Mo_ss_, where the subscript “ss” refers to the initials of the solid solution) as a ductile phase and another two intermetallic strengthening phases, i.e., Mo_3_Si (A15) and Mo_5_SiB_2_ (T_2_) [16]. These alloys show balanced mechanical properties from a reasonable microstructural design [17]. Figure 1 shows the Mo-rich portion of the 1600 °C isotherm of the Mo-Si-B phase diagram [16]. Although the BCC phase is significantly responsible for the ductility of the alloys, its volatile oxidation product of MoO_3_ always leads to a rapid mass loss upon high-temperature oxidation [18,19]. In addition, catastrophic oxidation of an entire breakdown of the bulk materials, usually called the “pest” phenomenon, often occurs during intermediate-temperature oxidation [19]. In order to obtain a better oxidation resistance without sacrificing the fracture toughness of the alloys, high Si and B contents and a considerable volume fraction of the BCC phase are simultaneously needed in Mo–Si–B alloys. In fact, it is impossible to achieve this requirement on the basis of the Mo–Si–B ternary phase diagram [16]. Once excess Si is added, large amounts of intermetallic phases have the chance to form and impair the fracture toughness of the alloy. Therefore, fourth-element alloying is desired and the chemical composition, phase constitution and microstructure should be carefully designed for these alloys. Recently, efforts have been made for the microstructural optimization of these alloys. Series of alloying elements have been tested and it was fortunately found that macroalloying of Ti could help achieve a novel phase equilibrium with both an adequate ductile BCC phase and a high Si concentration [20,21,22,23,24]. As a result, the alloys can possess a better balance between the room-temperature fracture toughness and high-temperature oxidation resistance. These findings opened a new door to alloy design and its application in the future.

In this review, the history of the development from Mo–Si–B to the novel Mo–Ti–Si–B-based alloys will be summarized. The alloy design strategy, including the phase equilibria and microstructure, will be analyzed. Mechanical properties, such as high-temperature strength, creep behavior and room-temperature fracture toughness, will be discussed. In addition, the oxidation behavior from intermediate to high temperatures will also be addressed. The authors hope that the present review might provide useful information for materials scientists and contribute to the progress of these novel ultra-high-temperature materials.

## 2. Why Use Mo–Ti–Si–B Alloys?

In a common Mo–Si–B alloy, the BCC phase is the only agent contributing to the room-temperature toughness. The other two intermetallic phases, A15 and T_2_, present the alloy with excellent strength and high-temperature creep resistance. It should be mentioned that the T_2_ phase is much more oxidation-resistant than A15 due to its B atoms. The existence of B favors the formation of a more passive film, called borosilicate (SiO_2_·B_2_O_3_), compared with the monolithic SiO_2_. It has been reported that with an increasing B content, the viscosity of SiO_2_ glass decreases drastically [25]. The borosilicate can spread out on the specimen surface through viscosity flow and protect the substrate [26,27]. Therefore, a combination of the ductile phase (BCC solid solution) and the oxidation-resistant strengthening phase (T_2_ compound) is preferred in Mo–Si–B alloys.

**Figure 2:**
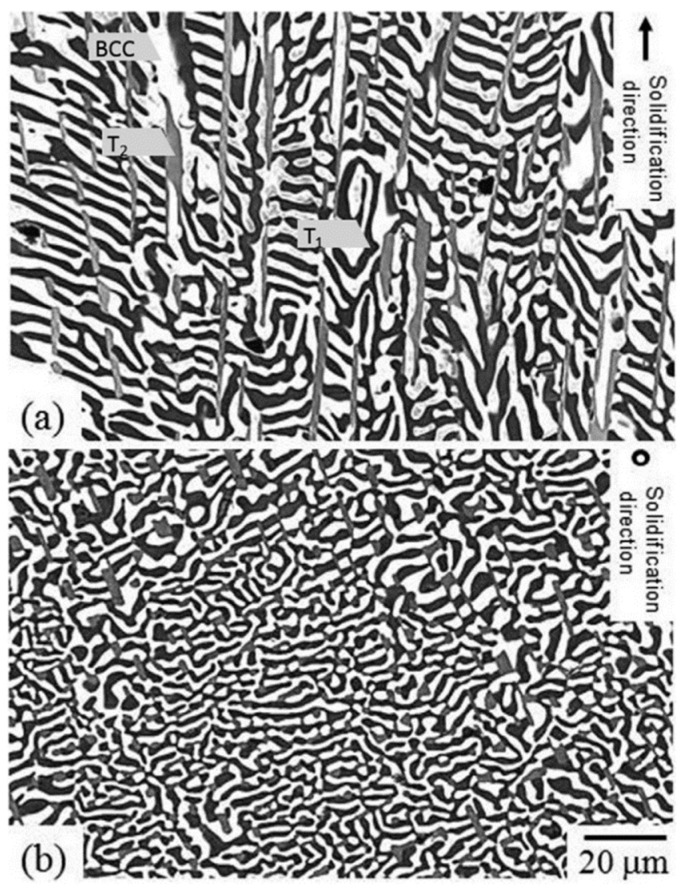
SEM-BSE images of the directionally solidified Mo–32.6Nb–19.5Si–4.7B (at.%) alloy: (**a**) longitudinal cross-section; (**b**) transversal cross-section [22]. (**a**,**b**) share the same scale bar.

In order to enlarge the BCC+T_2_ two-phase region, the effects of fourth-element alloying on the phase stability of the Mo–Si–B ternary alloys has been examined over the years, mainly focused on the refractory metal substitution of Mo [20,21]. For instance, the addition of Cr provides the possibility of establishing a BCC((Mo,Cr)_ss_)+T_2_((Mo,Cr)_5_SiB_2_) two-phase field [20]. More significantly, Nb had been chosen to enlarge the compositional span of BCC+T_2_ two-phase fields on the basis that a stable BCC(Nb_ss_)+T_2_(Nb_5_SiB_2_) duplex region can reside in a relatively wider composition range [20]. A BCC((Mo,Nb)_ss_)+T_1_((Mo,Nb)_5_Si_3_)+T_2_((Mo,Nb)_5_SiB_2_) three-phase eutectic microstructure could be further obtained, exhibiting an organized interwoven morphology through directional solidification (see Figure 2) [22].

Although the BCC((Mo,Nb)_ss_) phase and the intermetallics (T_1_ and T_2_) might play similar roles as γ and γ′ in Ni-based superalloys, respectively, the low interface energy of γ/γ′ is difficult to achieve in Mo–Nb–Si–B alloys [1]. Moreover, the most depressing outcome is the degraded oxidation resistance with Nb addition. Regardless of the fact that the oxidation product of Nb_5_O_3_ is not as volatile as MoO_3_, substitution of Nb for Mo drastically decreases the oxidation resistance of Mo-only intermetallic phases [28,29]. These quaternary alloys usually undergo rapid catastrophic oxidation (pest) at the intermediate temperature at ~800 °C [28].

**Figure 3:**
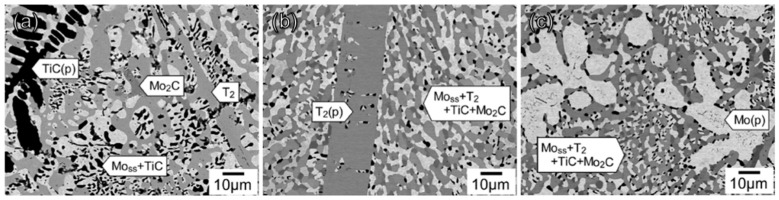
SEM-BSE images of microstructures for (**a**) Mo–5.0Si–10.0B–10.0Ti–10.0C; (**b**) Mo–6.7Si–13.3B–7.5Ti–7.5C; (**c**) Mo-5.0Si–10.0B–7.5Ti–7.5C (at.%) alloys after heat treatment at 1800 °C for 24 h [31].

Most recently, TiC-added Mo–Si–B alloys were designed by Miyamoto et al. [30]. Besides BCC and T_2_ phases, BCC+TiC, BCC+T_2_+TiC, BCC+T_2_+Mo_2_C and BCC+T_2_+TiC+Mo_2_C eutectics were formed [30]. Figure 3 shows the microstructure of some typical TiC-added Mo–Si–B alloys after annealing at 1800 °C for 24 h [31]. The primary phases can vary from TiC to T_2_ and to BCC with slight changes in Si, B and TiC contents.

In these alloys, TiC can act as an excellent strengthening phase not only due to its high strength and high melting points, but also its low density. It should also be mentioned that the primary TiC tends to precipitate in priority to the BCC+TiC eutectic due to the high melting point of TiC (3140 °C), which is much higher than that of pure Mo (2623 °C). As a result, the continuity of the BCC matrix is highly facilitated in these TiC-added Mo–Si–B alloys. In fact, it has been reported that the fracture toughness increases with a higher volume fraction of the toughening phase but decreases when its scale decreases [17]. Therefore, a continuous BCC matrix is always favored to improve the fracture toughness of Mo–Si–B alloys [17]. Moreover, the fractographic results of river patterns on the fracture surface of the TiC phase suggest an extra-toughening mechanism compared with the T_2_ phase, which shows a flat and smooth fracture surface [31]. In this view, besides the strengthening effect of TiC, the as-cast structure of a continuous BCC matrix highly contributes to the ductility of these alloys. The TiC-added Mo–Si–B alloys exhibit superior mechanical properties, including high room-temperature toughness and good high-temperature creep resistance [31,32,33,34,35]. However, in order to suppress the formation of extra (boro)silicides and reserve enough BCC phase to ensure its adequate toughness, high Si content, which is responsible for excellent oxidation resistance, is not permitted in these alloys. In consequence, the trade-off between mechanical properties and oxidation resistance still remains unsolved.

Therefore, one important task to balance the mechanical properties and oxidation resistance is to preserve as much BCC phase as we can and simultaneously increase the Si content. In this case, oxidation resistance can be improved since SiO_2_-based passive films will more easily form assisted by the high Si content, and the fracture toughness is able to be guaranteed due to sufficient BCC phase. To realize this goal, alloying elements that can stabilize the BCC phase are preferred. Dimiduk and Perepezko [8] summarized some commonly used transition elements that dissolve within the three base phases (BCC, T_2_, A15) in a ternary Mo–Si–B alloy or that introduce new phases existing in higher-order spaces. Impressively, they pointed out that Ti, Zr, Hf, V, Nb, Ta, Cr and W could act as BCC stabilizers for Mo–Si–B alloys. Among these alloying elements, Ti became the most satisfactory one not only due to its relatively low density but also due to the formation of a D8_8_-structured Ti_5_Si_3_ phase upon macroalloying [23,36,37]. The D8_8_ phase has a high melting point of 2130 °C and a low density of 4.32 g/cm^3^ [38]. Besides its high values of hardness and elastic modulus, the creep strength of this D8_8_ compound is much higher than that of other structural intermetallics with cubic or hexagonal crystal structures, such as NiAl, FeAl or Ti_3_Al [38]. In addition, D8_8_-Ti_5_Si_3_ possesses good oxidation resistance without pesting [39,40]. For the above reasons, the incorporation of the D8_8_ phase into Mo–Si–B alloys may contribute to a better comprehensive performance. In other words, quaternary Mo–Ti–Si–B alloys should be superior compared with their ternary ancestors (Mo–Si–B alloys). The lattice structure data of some typical compounds in Mo–Ti–Si–B alloys can be found in Table A1 in the Appendix A.

## 3. Microstructure of Mo–Ti–Si–B Alloy Systems

### 3.1. Thermal Dynamic Calculation

**Figure 4:**
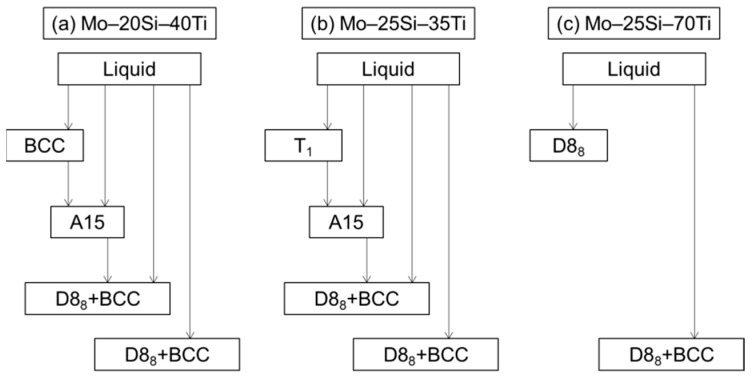
Brief flow chart of the solidification sequence for (**a**) Mo–20Si–40Ti, (**b**) Mo–25Si–35Ti and (**c**) Mo–25Si–70Ti alloys (at.%).

The phase constitution of Mo–Ti–Si–B systems has been systematically investigated during the past 20 years [36,37,41,42]. In order to gain better understanding of this quaternary alloy, the thermodynamic description of the ternary Mo–Ti–Si system was firstly studied with the combination of CALPHAD (Calculation of Phase Diagram) approaches and experimental observation [41]. Typical solidification sequences were determined for the metal-rich Mo–20Si–40Ti, Mo–25Si–35Ti and Mo–25Si–70Ti alloys (at.%). For Mo–20Si–40Ti, the primary phase during solidification is BCC((Mo,Si,Ti)_ss_). After that, the A15 phase forms through the peritectic ridge of L+(Mo,Si,Ti)_ss_→(Mo,Ti)_3_Si and is then partially decomposed by the reaction of L+(Mo,Ti)_3_Si→(Ti,Mo)_5_Si_3_(D8_8_)+(Mo,Si,Ti)_ss_. Finally, the eutectic microstructure arises via L→(Ti,Mo)_5_Si_3_+(Mo,Si,Ti)_ss_. For Mo–25Si–35Ti, the primary phase is (Mo,Ti)_5_Si_3_(T_1_). Then the peritectic ridge of L+ (Mo,Ti)_5_Si_3_→(Mo,Ti)_3_Si takes place and the rest of the solidification path is the same as that of Mo–20Si–40Ti. These alloys are thereby composed of BCC, A15 and D8_8_ phases. However, for Mo–25Si–70Ti, the primary phase becomes D8_8_ and then the (Ti,Mo)_5_Si_3_+(Mo,Si,Ti)_ss_ eutectic forms. No A15 comes out. (The version and principle calculation parameters can be found in Ref. [41]). Figure 4 illustrates a brief flow chart of the solidification sequence for these three alloys. It is suggested that with more Mo atoms replaced by Ti atoms in the metal-rich Mo–Ti–Si system, one of the base phases of A15 in traditional Mo–Si–B alloys would be gradually substituted by the D8_8_ phase.

**Figure 5:**
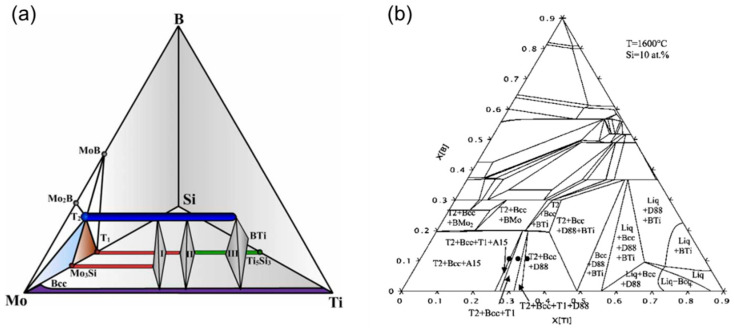
(**a**) Schematic isothermal tetrahedron displaying the phase relationships among BCC, T2, A15, T1 and D88 on the metal-rich side of the Mo–Ti–Si–B quaternary system at 1600 °C; (**b**) Isothermal section of Mo–Ti–Si–B at 1600 °C with Si = 10 at.% [36].

Based on the above results, multiphase equilibria in the metal-rich region of Mo–Ti–Si–B system were carefully investigated [36,37]. The key alloys, based on the constituent phases of BCC, T_2_, T_1_, D8_8_ and A15, were selected with the guidance of the phase diagram calculated from thermodynamic modeling using the commercially available software package Pandat (Version 7). Figure 5a [36] demonstrates the calculated Mo–Ti–Si–B isothermal tetrahedron at 1600 °C, but only the phase relationships involving BCC, T_2_, T_1_, D8_8_ and A15 are schematically shown considering the current focused interests. The widths of two horizontal lines represent two narrow single-phase zones of T_2_ (dark blue) and BCC (purple).

Here I, II and III represent three four-phase regions of BCC+A15+T_2_+T_1_, BCC+T_2_+T_1_+D8_8_ and BCC+T_2_+D8_8_+BTi. Spaces between these regions are four different three-phase windows, i.e., A15+T_2_+T_1_ and BCC+A15+T_2_ on the left side of Region I, BCC+T_2_+T_1_ between Region I and Region II, and BCC+T_2_+D8_8_ between Region II and Region III. Figure 5b [36] manifests the slice of 10 at.% Si taken from the tetrahedron in Figure 5a. The above three-phase windows, except for the A15+T_2_+T_1_ that does not include the ductile BCC phase, can be clearly distinguished at the Mo-rich corner. It should be noted that the three-phase equilibrium of BCC+T_2_+T_1_ is much narrower than the other two windows. It is also revealed that this three-phase equilibrium is more stable at low temperatures than it is at high temperatures. Therefore, if the target microstructure is BCC+T_2_+T_1_, the compositional requirement will be strict and the desired alloy has less tolerance at high temperatures than at low temperatures. Recall that in the view of microstructure optimization for the Mo–Ti–Si–B alloys, the multi-phase equilibria of BCC+T_2_+A15, BCC+T_2_+T_1_, BCC+T_2_+D8_8_, BCC+T_2_+A15+T_1_ and BCC+T_2_+T_1_+D8_8_ are combinations of both the ductile BCC phase and the strengthening intermetallic compounds, which is promising for the improved mechanical properties. For instance, Mo–37.5Ti–18Si–9B locates in the BCC+T_2_+D8_8_ field whereas Mo–28.5Ti–18Si–9B and Mo–32.5Ti–18Si–9B do so in the fields of BCC+T_2_+A15+T_1_ and BCC+T_2_+T_1_+D8_8_, respectively (at.%) [37]. Additionally, the microstructures based on the three-phase equilibria of BCC+T_2_+T_1_ and BCC+T_2_+D8_8_ are expected to afford better oxidation resistance than those on BCC+A15+T_2_ due to a higher Si concentration. In other words, macroalloying of Ti stabilizes the BCC phase to the high Si content, where the A15 phase can be totally suppressed as in the Mo–Ti–Si system.

**Figure 6:**
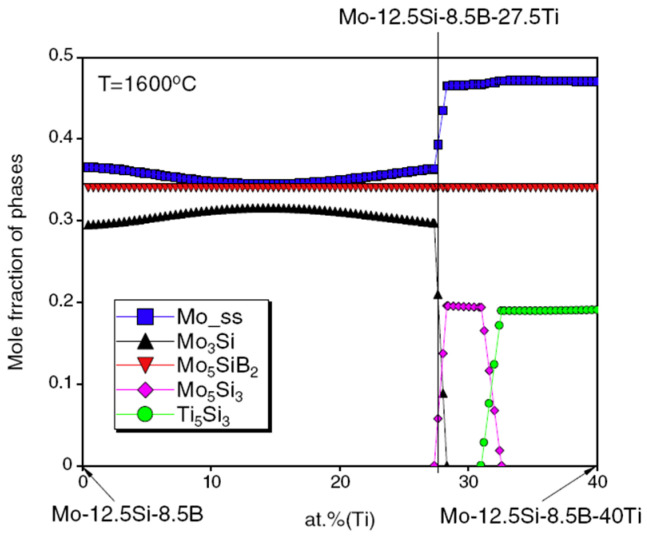
Calculated mole fractions of phases in (79 − *x*)Mo–xTi–12.5Si–8.5B (at.%) at 1600 °C as a function of Ti content [42].

The effects of macroalloyed Ti on the phase equilibria of Mo–Ti–Si–B alloys are further studied focused on the ones with the Si and B contents of 12.5Si–8.5B and 9Si–8B (at.%) [42,43]. For instance, Figure 6 demonstrates the calculated mole fractions of constituent phases in (79 − *x*)Mo–*x*Ti–12.5Si–8.5B (0 ≤ *x* ≤ 40, at.%) at 1600 °C [42]. It is clearly seen that with increasing Ti content, the phase equilibrium of this alloy changes in the sequence of “BCC+A15+T_2_”, “BCC+A15+T_2_+T_1_”, “BCC+T_2_+T_1_”, “BCC+T_2_+T_1_+D8_8_” and “BCC+T_2_+D8_8_”. In addition, a considerable number of Ti atoms can be dissolved into the BCC, A15, T_1_ and T_2_ phases. The phase equilibrium of the 9Si–8B alloy has a similar tendency that is not shown here. Apparently, Ti content acts as one of the most crucial parameters for the microstructure development in the Mo–Ti–Si–B quaternary systems. With this guidance, the target phase constitution, or the desired microstructure, may be successfully tailored.

### 3.2. Experimental Characterization

**Figure 7:**
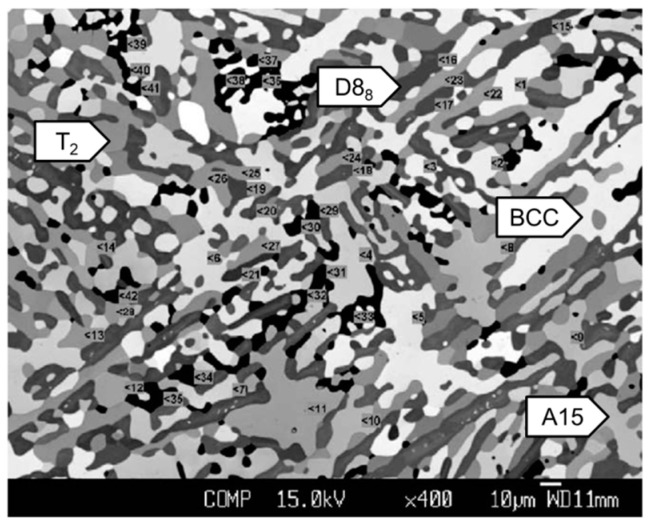
BSE image of the microstructure of the Mo–12.5Si–8.5B–27.5Ti (at.%) alloy after annealing at 1600 °C for 150 h, showing BCC (bright), A15 (gray), T_2_ (darker gray) and D8_8_ (black) phases [42].

In order to obtain the BCC-containing Mo–Ti–Si–B alloys, two kinds of alloys with the compositions of Mo–27.5Ti–12.5Si–8.5B and Mo–29Ti–9Si–8B (at.%) were designed and prepared based on the thermodynamic calculation [42,43]. They should possess the phase equilibria of BCC+A15+T_2_+T_1_ and BCC+T_2_+T_1_, respectively, if the thermodynamic prediction holds true. These alloys were obtained by arc-melting and annealed at 1600 °C for 150 h in vacuum to reach phase equilibrium. Figure 7 [42] shows the representative microstructure of the annealed Mo–27.5Ti–12.5Si–8.5B, where the white, light grey, dark grey and black phases in these SEM-BSE images refer to the BCC, A15, T_2_ and D8_8_ phases, respectively. A similar microstructure had been found for Mo–29Ti–9Si–8B, except for the absence of A15 [43].

Contrary to the thermodynamic predictions as stated in Section 3.1, the desired T_1_ phase appeared in neither of the alloys [43]. Instead, a small amount of the D8_8_ phase was observed in both of them. The reason for the discrepancy between the calculation and the experimental results has not been clarified yet, but the strong thermal stability of the D8_8_ phase may give it the chance to form in the alloys. It should be mentioned that as predicted by thermodynamic calculation, the high solubility of Ti in every phase was observed, which led to a density reduction of nearly 18% as compared with the reference Mo–Si–B alloy [44]. The density reduction is one of the most attractive advantages for the macroalloying of Ti over other BCC stabilizers [23].

For microstructure optimization, the composition dependence of the phase constitution of the alloys was further studied. Before the detailed study on Mo–Ti–Si–B quaternary alloys, the Mo–Ti–Si system was first analyzed. In order to reduce the lateral dimensions of constituent phases, which is required for a better oxidation resistance [26], the eutectic and eutectoid Mo–Ti–Si alloys were designed and studied by Schliephake et al. [45]. These two alloys were composed of BCC+D8_8_ and BCC+D8_8_+T_1_, respectively. The alloy compositions were selected based on the simulation results, especially focusing on two distinct reactions of (1) L→BCC+D8_8_ and (2) A15→BCC+T_1_. In their study, the composition of the eutectic alloy was decided to be Mo–52.8Ti–20.0Si whereas that of the eutectoid one was Mo–34Ti–21Si (at.%). Regarding these two alloys as references, Obert et al. [46] further studied the other two metal-rich Mo–Ti–Si alloys with tailored eutectic-eutectoid microstructures comprising minor volume fractions of primary solidified BCC. For the eutectic structure, it seems that both phases presented as laterally expanded interconnecting networks, which is not possible to assign to the matrix-forming phase [47]. In addition, all of these alloys possess fine-scaled microstructures.

**Figure 8:**
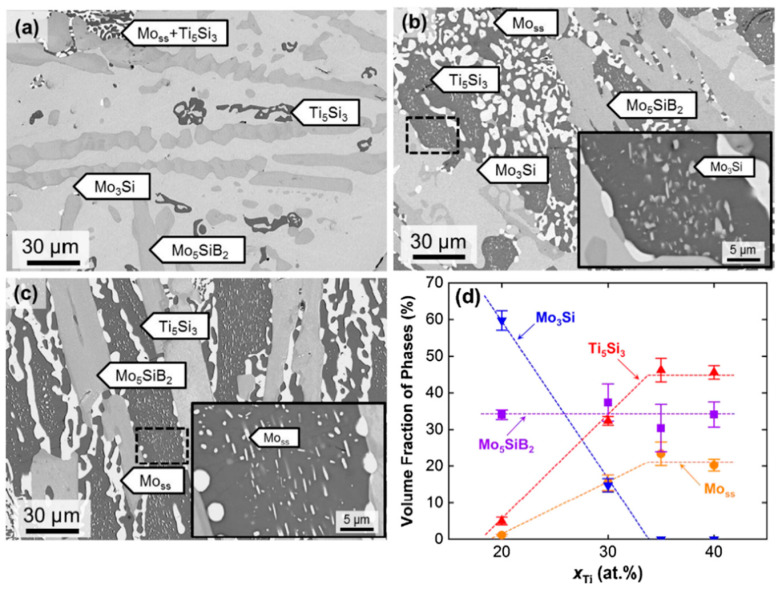
Microstructure of (**a**) Mo–20Ti–20Si–10B, (**b**) Mo–30Ti–20Si–10B and (**c**) Mo–40Ti–20Si–10B alloys after annealing at 1600 °C for 24 h. The inserts are high-magnification images in dashed squares of the corresponding alloys. (**d**) Experimental measured volume fractions of constituent phases in (70 − *x*)Mo–xTi–20Si–10B (at.%) alloys (20 ≤ *x* ≤ 40) [48].

Except for the formation of the T_2_ phase, microstructure evolution in the Mo–Ti–Si–B quaternary alloys with increasing Ti content does not seem to make much difference compared with that of Mo–Ti–Si alloys. Zhao et al. [48] designed and investigated three kinds of cast Mo–Ti–Si–B alloys, i.e., Mo–20Ti–20Si–10B, Mo–30Ti–20Si–10B and Mo–40Ti–20Si–10B (at.%). These alloys contained the same Si and the same B contents while having a trade-off between Mo and Ti. Phase constitution of the as-cast microstructure varied from BCC+D8_8_+T_2_+T_1_+A15, to BCC+D8_8_+T_2_+A15, and to BCC+D8_8_+T_2_ with increasing Ti concentration. The situation slightly changed after high-temperature annealing (1600 °C for 24 h). Figure 8 [48] presents the microstructures of the annealed alloys.

It should be noted that the T_1_ phase vanished in the Mo–20Ti–20Si–10B alloy and apparent microstructure coarsening took place in the eutectic structure for all alloys. Figure 8d, which summarizes the volume fractions of constituent phases in these alloys, expresses an overall trend that the volume fractions of the BCC phase and D8_8_ phase increase with increasing Ti concentration whereas that of the A15 phase has the opposite tendency. This result brings us the good news that even for the complex quaternary system, the phase constitution and volume fraction of each phase can be easily tailored by varying the Ti content. According to the criterion for microstructure optimization as discussed above, the Mo–40Ti–20Si–10B alloy of BCC+D8_8_+T_2_ may have the most promising properties among the three compositions regarding the existence of enough BCC phase and the absence of low-Si A15 phase. In this regard, under the conditions of a well-designed alloy system, an appropriate microstructure can be achieved for the Mo–Ti–Si–B quaternary system.

**Figure 9:**
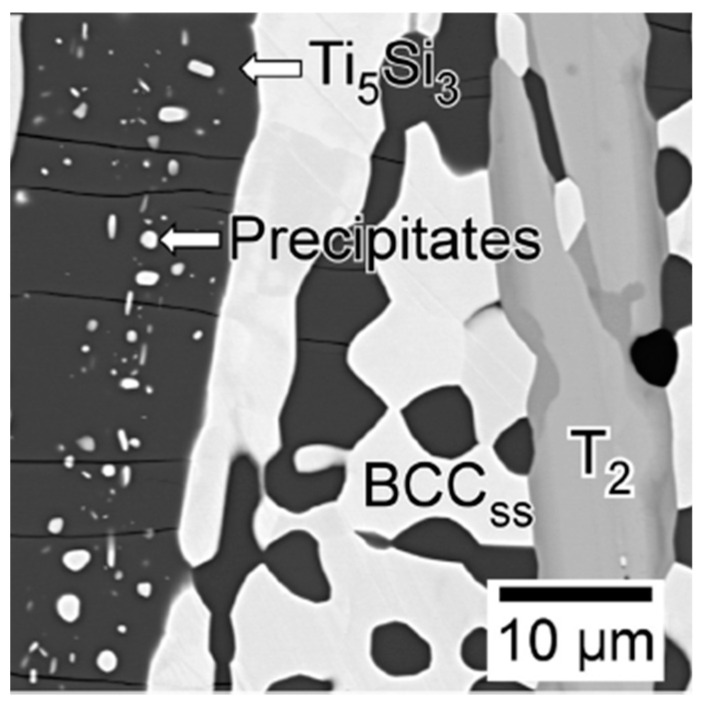
Microstructure of Mo–50Ti–14Si–6C–6B (at.%) alloy annealed at 1500 °C for 24 h [53].

It is the formation of the D8_8_ phase in Mo–Ti–Si–B alloys that makes them different from their Mo–Si–B counterparts. High strength, low density and superior oxidation resistance of the D8_8_ phase can no doubt give the alloy better high-temperature performance [38,39,40,49,50]. The intrinsic brittleness of intermetallic compounds, however, becomes the next challenge for these D8_8_-containing alloys. In these alloys, the primary D8_8_ grains are always largely elongated along the solidification direction due to a preferential growth along the *c*-axis [35]. Since it is a highly ordered phase, it has high susceptibility to crack formation. Microcracks are often observed across the longitudinal directions of these alloys [35]. According to previous studies [38], these microcracks should be initiated by the local density of microvoids, residual stress fields and the anisotropy of the linear coefficient of thermal expansion (CTE). The CTE of D8_8_-Ti_5_Si_3_ is 5.058 × 10^−6^ K^−1^ along the *a*-axis and 22.197 × 10^−6^ K^−1^ along the *c*-axis [51], yielding a thermal expansion anisotropy of ∼4.4 at room temperature. The cooling process leads to the anisotropic shrinkage of this phase, and gives rise to microcracking perpendicular to its *a*-axis. Recall that the D8_8_-Ti_5_Si_3_ compound in the Mo–Ti–Si–B system is always off-stoichiometric with a considerable amount of Mo dissolved. Upon annealing, Mo-rich BCC particles can precipitate within the primary D8_8_ grains by the diffusion of supersaturated Mo atoms [35,48,52,53]. The BCC precipitates (see Figure 9 [53]) are promising for enhancing the toughness of the alloy by inhibiting the propagation of microcracks within the D8_8_ matrix.

**Figure 10:**
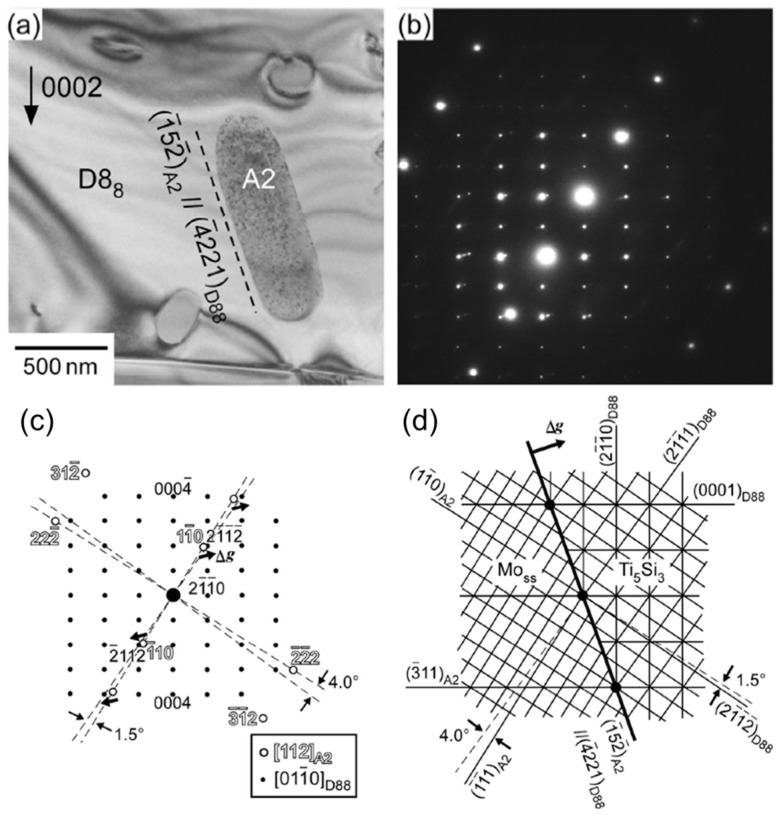
(**a**) A bright-field image; (**b**) corresponding diffraction pattern; (**c**) a key diagram of the diffraction pattern and (**d**) a schematic of the corresponding two-dimensional lattice plane matching for the BCC precipitates and the D8_8_ matrix of the Mo–50Ti–14Si–6C–6B (at.%) alloy [53].

Hatakeyama et al. [53] analyzed the orientation relationship between BCC precipitates and the D8_8_ matrix of the Mo–50Ti–14Si–6C–6B (at.%) alloy (see Figure 10). They found that the faceted interfaces between BCC and D8_8_ meet an edge-to-edge [54] matching that produces low energy and are stochastically favored during solid-state precipitation. Thanks to these thermally stabilized BCC precipitates and their low-energy interfaces, the toughness of these alloys may be improved by the crack bridging or crack deflection mechanisms if the size and distribution of the precipitates are carefully tailored through an appropriate annealing process.

## 4. Properties of the Mo–Ti–Si–B Alloy System

### 4.1. Mechanical Properties

As one of the representative phases in the Mo–Ti–Si–B system, the deformation behavior of the D8_8_ grains plays a significant role in the mechanical properties. Umakoshi and Nakashima [55] investigated the high-temperature deformation behavior of Ti_5_Si_3_ single crystals several decades ago. It was found that the yield stress had strong orientation dependence and numerous twins were observed in the deformed specimens since the deformation of Ti_5_Si_3_ crystals was mainly controlled by twinning with a $\{10\bar{1}2\}\langle 10\bar{1}\bar{1}\rangle$ system. After that, Kishida et al. [56] carefully studied the plastic deformation mechanism of this D8_8_ phase. It was proved that prismatic slip, pyramidal slip and twinning, depending on the loading axis, were operative at temperatures above 1300 °C.

**Figure 11:**
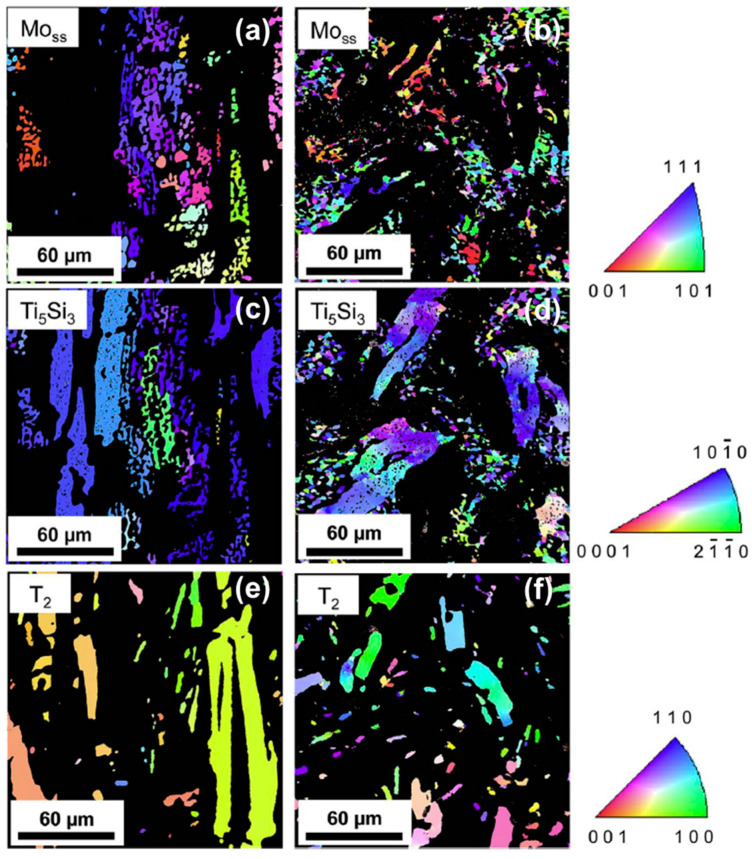
IPF maps of the main constitution phases (**a**,**c**,**e**) before and (**b**,**d**,**f**) after high-temperature deformation [35].

In the multiphase Mo–Ti–Si–B-based alloys, despite its off-stoichiometry, the D8_8_ phase is deformable at high temperatures. Generally, these alloys exhibit excellent high-temperature strength. Zhao et al. [35] investigated the compressive deformation behavior of the D8_8_-containing Mo–30Ti–17Si–10C–5B (at.%) alloy at 1500 °C, and found it impressive that the ultimate compressive strength of this alloy could exceed 500 MPa. In addition, this alloy possessed good deformability at high temperatures and macrocracks were hardly seen on the specimen surface even if the total plastic strain (true strain) reached 79%. Figure 11 [35] manifests the Inverse Pole Figure (IPF) maps of the BCC ((a,b)), D8_8_ ((c,d)) and T_2_ ((e,f)) phases before ((a,c,e)) and after ((b,d,f)) high-temperature compression tests.

It was revealed that both the BCC and D8_8_ phases displayed some ductility at this testing temperature. Recall that the ductile-brittle transition temperature (DBTT) of the D8_8_ is about 1200 °C [57]. In this regard, the dynamic recovery and recrystallization can easily take place, not only in BCC but also in D8_8_ during deformation at 1500 °C. As it can be seen in Figure 11b,d, numerous grain boundaries were generated within the isolated BCC and D8_8_ phases. In contrast, The DBTT of T_2_ is approximately 1500 °C [57], which reaches the same level as the testing temperature. Therefore, the T_2_ phase could still act as the strengthening phase without dynamic recrystallization, which was responsible for the excellent high-temperature strength of the alloy.

**Figure 12:**
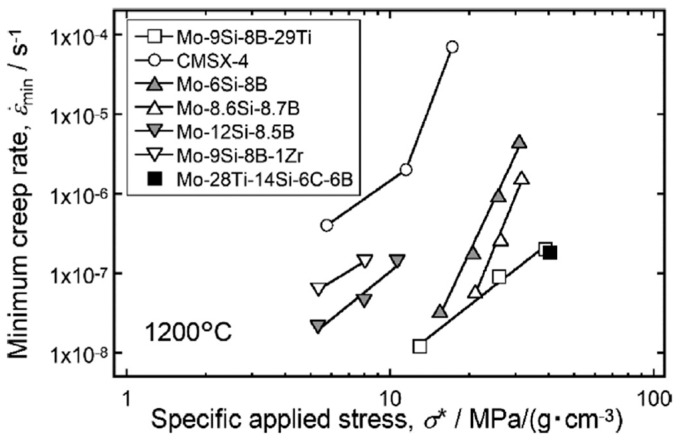
Double logarithmic plot of the minimum creep rate against applied stress at 1200 °C and 300 MPa of Mo–Ti–Si–B-based alloys compared with those of other Mo–Si–B alloys and the single-crystalline Ni-based superalloy CMSX-4 [34].

As for high-temperature structural materials, enhancing the high-temperature creep resistance of these novel Mo–Ti–Si–B alloys is one of the most crucial issues in their engineering application. Schliephake et al. [43] tested the compressive creep behavior at 1200 °C and 1300 °C of two Mo–Ti–Si–B alloys (Mo–27.5Ti–12.5Si–8.5B and Mo–29Ti–9Si–8B, at.%) for the first time. Rather than the pronounced microstructural changes during creep such as rafting in single crystalline Ni-based superalloys, it was found that the aforementioned two alloys possessed relatively stable microstructures and near-steady-state creep conditions. The stress exponents appeared to be between 3 and 3.5, indicating a dislocation climb-controlling creep mechanism for both of the alloys. It should be mentioned that the strain rate of monolithic Ti_5_Si_3_ at 1200 °C was reported to be approximately 10^−6^ s^−1^ at 100 MPa with a stress exponent of 3 [38]. Since the strain rate of pure Ti_5_Si_3_ is two orders of magnitude higher than that of the multiphase Mo–Ti–Si–B alloys, the D8_8_ phase is believed to participate in the creep process. The activation energy of Mo–29Ti–9Si–8B was estimated to be comparable to that of Mo self-diffusion, suggesting that bulk diffusion of Mo atoms in the BCC phase might play the major role in creep deformation of the alloy. Hereafter, Azim et al. [58] further studied the creep behavior of several Mo–Ti–Si–B alloys with different phase constitutions in the temperature range of 1100 °C–1300 °C. Unlike the T_2_ phase, which hardly crept at the testing temperature, the intermetallic phases of A15 and D8_8_ could be deformed during creep, in which many dislocations were observed after the tests. Most recently, Hatakeyama et al. [34] tested the Mo–28Ti–14Si–6B–6C (at.%) alloy at 1200 °C–1300 °C and 100 MPa–300 MPa. Based on the experimental data, they yielded the apparent activation energy of this alloy at 300 MPa to be 639 kJ/mol. Since this value was close to the activation energy of the monolithic D8_8_ compound (620 kJ/mol–640 kJ/mol [59]), a significant contribution from the D8_8_ phase to the creep deformation of the alloys can be understood. Comparison has been made for the creep behavior at 1200 °C between this alloy and other common high-temperature materials. Figure 12 [34] summarized the minimum creep rate of different alloy systems as a function of density-normalized applied stress, indicating the excellent creep resistance of this Mo–28Ti–14Si–6B–6C alloy.

Note that the testing temperature (1200 °C) approaches the solvus temperature of the γ′ phase in a single-crystal Ni-based superalloy CMSX-4; the creep strength of CMSX-4 is markedly inferior to those of the Mo–Si–B- or Mo–Ti–Si–B-based alloys [60]. Moreover, good news comes that the newly developed Mo–Ti–Si–B or TiC-added Mo–Ti–Si–B alloys manifested superior creep resistance to that of the well-studied traditional Mo–Si–B alloys, partially attributed to the solid-solution strengthening by larger amounts of solute elements in the BCC phase.

Hatakeyama et al. [34] also briefly investigated the room-temperature fracture toughness of the above TiC-added Mo–Ti–Si–B alloy, which was determined to be (12.8 ± 1.2) MPa (m)^1/2^ according to their three-point bending tests. It was also proved that the Si content in the BCC phase, which could be easily adjusted by varying the annealing temperature, played a significant role in the fracture toughness of the alloy. Generally, a lower Si content results in a better fracture toughness for alloys with identical volume fractions of constituent phases.

### 4.2. Oxidation Behavior

As it is widely understood, refractory metal-based alloys and composites always suffer from poor oxidation resistance at elevated and high temperatures because of the volatile oxidation products from the refractory metal elements. The novel Mo–Ti–Si–B alloys contain a considerable amount of Mo, whose main product, MoO_3_, starts to severely volatilize at 750 °C [61]. The suppression of the vitalization of MoO_3_ should be a crucial issue for the enhancement of the oxidation resistance of these alloys. The first report concerning the oxidation behavior of Mo–Ti–Si–B alloys was presented by Azim et al. [62] around 10 years ago. They tested the oxidation behavior of Mo–29Ti–9Si–8B and Mo–27.5Ti–12.5Si–8B (at.%) alloys in the temperature range between 820 °C and 1300 °C. It was revealed that the oxidation kinetics became totally different at intermediate and high temperatures, and both of the alloys showed rapid mass loss at 820 °C and 1000 °C [62].

**Figure 13:**
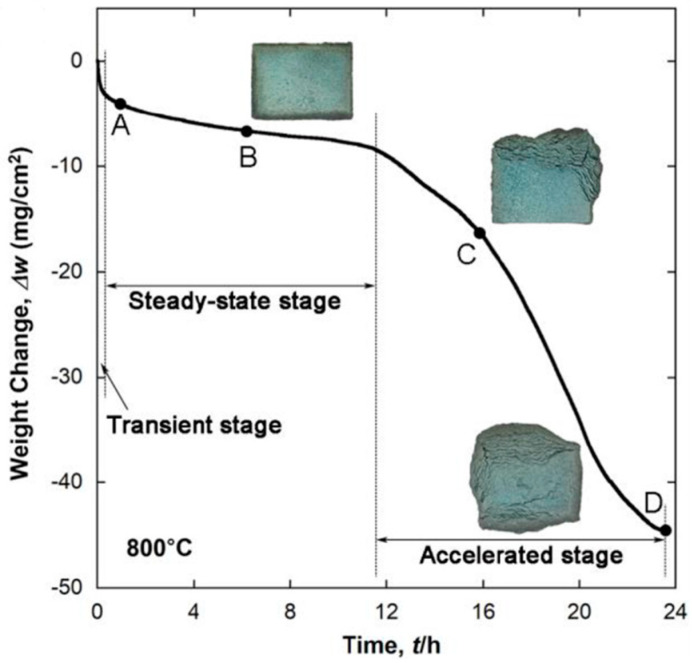
Isothermal oxidation curve of the Mo–30Ti–17Si–5B–10C (at.%) alloy oxidized at 800 °C, along with the appearance of the oxidized specimens [66].

Pest degradation occurred at an intermediate temperature range (below 1000 °C), indicated by the rapid mass loss upon oxidation. This is similar to the oxidation behavior of some molybdenum silicides [63,64] and the Mo–Si–B ternary alloys [19,65]. Fortunately, the oxidation resistance at higher temperatures (above 1000 °C) seems not to be disappointing. The oxidation scale formed at high temperatures is reported to be composed of four layers, i.e., an outermost TiO_2_-predominant layer, a duplex layer composed of crystalline-TiO_2_ and amorphous-SiO_2_, an interlayer mainly consisting of MoO_2_ and an inner oxidation zone after long-time oxidation. A significant reduction in the initial evaporation rate of MoO_3_ was realized in these quaternary alloys beyond 1000 °C compared with the referring Mo–Si–B alloys. After that, Nan et al. [66] further analyzed the oxidation kinetics of a TiC-added Mo–Ti–Si–B alloy with the nominal composition of Mo–37Ti–17Si–5B–10C (at.%) during oxidation from 700 °C to 1100 °C. Except for a short-term weight gain before switching to weight loss at 700 °C, the specimens exhibited continuous weight loss at all the other testing temperatures, and the most rapid weight-loss rate appeared at 800 °C as a result of the troublesome pest phenomenon (see Figure 13 [66]). At 900 °C and 1000 °C, the weight loss could be fitted to parabolic rate kinetics with the rate of 0.49 mg^2^/cm^4^/h and 3.51 mg^2^/cm^4^/h, respectively [66].

According to the aforementioned results, it is reasonable to reach the common-sense conclusion that the suppression of pest degradation should be the next task for developing novel Mo–Ti–Si–B-based alloys. To gain a better understanding of the pest phenomenon, especially the influence of phase constitution and alloy composition on it, Schliephake et al. [45] tested the BCC+D8_8_ alloy (eutectic alloy) of Mo–52.8Ti–20Si and the BCC+D8_8_+T_1_ alloy (eutectoid alloy) of Mo–34Ti–21Si (at.%) under isothermal and cyclic oxidation conditions. It was impressive that the eutectic alloy was pest-resistant, successfully protected by a homogeneous layer of a mixture of TiO_2_ and SiO_2_. In contrast, the eutectoid one experienced catastrophic degradation and was completely oxidized. It should be mentioned that the amount of non-protective BCC phase was almost equivalent in these two alloys, and the lateral dimension of the BCC phase was even smaller in the catastrophically failed eutectoid one. In their opinion, the volume fraction of BCC phase and its size scale were not decisive in the formation of protective oxide scale. After that, Obert et al. [46] further studied the oxidation behavior of several D8_8_-containing Mo–Ti–Si systems, which should be helpful when it comes to the more complex Mo–Ti–Si–B quaternary system. They proposed that the threshold for the pest-resistant alloys lay in a nominal Ti content of minimum 43 at.% and an average Ti content in BCC of minimum 35 at.%. Then, they proved that the Ti content was one of the most significant influencing factors contributing to the intermediate-temperature oxidation resistance [47]. Moreover, Lu et al. [67] pointed out that the D8_8_-containing alloys could achieve good oxidation resistance at up to 900 °C benefiting from the self-healing ability where the amorphous SiO_2_ seals the clearances between the TiO_2_ grains.

**Figure 14:**
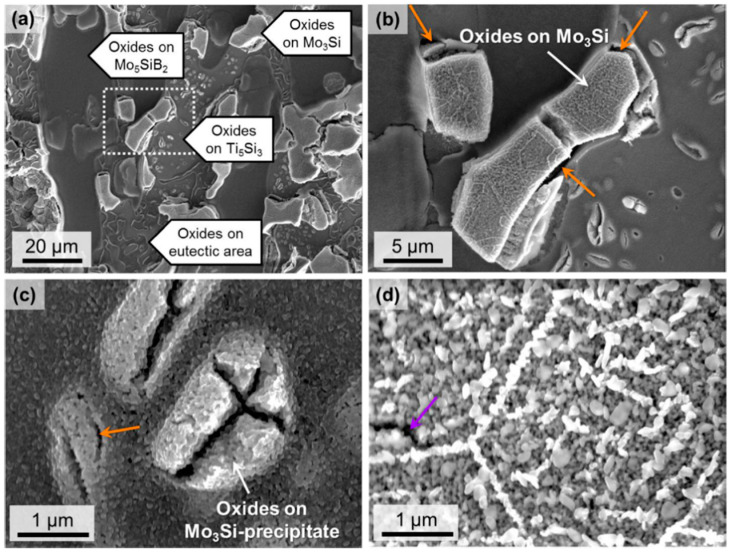
(**a**) Surface morphology of Mo–30Ti–20Si–10B (at.%) oxidized at 800 °C for 5 min. (**b**) is the enlarged image of the dashed square in (**a**). (**c**) shows the appearance of A15 precipitates within D8_8_ after oxidation. (**d**) is a high-resolution image of surface oxides on primary A15 [48]. All of them are SEM-SE images.

Phase constitution and phase equilibria, which also largely rely on the Ti content, are no doubt more complicated in Mo–Ti–Si–B alloys. Zhao et al. [48] carefully studied the series of Mo–(70 − *x*)Ti–20Si–10B (*x* = 20, 30, 40; at.%) quaternary alloys with different phase constitutions as discussed in above mentioned section. It was interesting to find that the A15 phase manifested the worst oxidation resistance especially at the initial stage of oxidation at 800 °C; its oxidation products were full of microcracks and were totally porous (see Figure 14 [48]).

**Figure 15:**
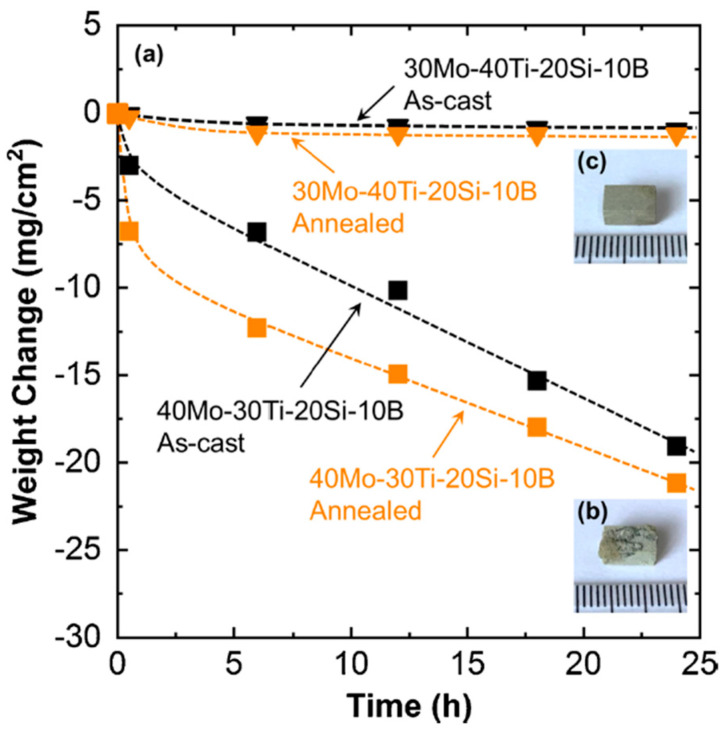
(**a**) Specific weight change of Mo–30Ti–20Si–10B and Mo–40Ti–20Si–10B (at.%) alloys oxidized at 800 °C. Black points refer to the data of as-cast alloys and orange ones refer to annealed alloys. (**b**,**c**) are the respective micrographs of the annealed specimens after oxidization at 800 °C for 24 h [48].

The pest degradation often occurs in alloys containing a considerable amount of the A15 phase. In these alloys, the swelling of the oxide products with porous structure is one of the main reasons leading to the pest phenomenon. In addition, due to its porous nature, oxygen can easily penetrate through the scale and give rise to a thick and high-oxygen-containing internal zone. As a result, catastrophic degradation rapidly proceeds. Fortunately, pest phenomenon can be successfully prevented if the volume fraction of A15 phase is suppressed. For the A15-free Mo–Ti–Si–B alloy, of course with a high Ti content, a thin and stable passive film can form and protect the specimens, resulting in a negligible mass loss during long-term oxidation at 800 °C (see Figure 15 [48]). Therefore, it is the high Ti content accompanied by the simple phase constitution (BCC+D8_8_+T_2_) that brings about the pest-resistant novel Mo–Ti–Si–B alloys [48,52].

The oxidation resistance of the alloys can be further enhanced through alloying. For instance, Al and Cr are proven to be effective to improve the oxidation resistance of D8_8_-incorporated Mo–Ti–Si–B, TiC-added Mo–Ti–Si–B and other Mo-based alloys [27,68,69,70]. The passive effect is mainly attributed to the formation of Cr_2_(MoO_4_)_3_ and Al_2_(MoO_4_)_3_ complex oxides at intermediate temperatures and Cr_2_O_3_ and Al_2_O_3_ at high temperatures. In particular, it should be noted that the replacement of Cr (at least up to 10 at.%) for Mo hardly changes the phase constitution of the alloys [69], allowing it to preserve the advantages in the microstructure and thereby in the mechanical properties. That is to say, the presence of a high-volume fraction of the BCC phase, which contributes to the fracture toughness, is feasible in these Cr-added alloys. Besides Al and Cr, microalloying of rare earth elements might also make sense. In previous studies, it was revealed that the addition of Y, La and Ce could result in the improved oxidation resistance of Mo–Si–B and Nb–Si alloys [71,72,73,74,75,76]. Since minor addition of rare earth elements usually brings about remarkable outcomes, the simultaneous optimization of microstructure and oxidation resistance of the newly developed Mo–Ti–Si–B-based alloys, we believe, will also be realized by the rare earth element modification.

## 5. Outlook

For a long time, the coexistence of a high volume fraction of BCC phase and intermetallic compounds of high Si content was seldom possible in traditional ternary Mo–Si–B alloys, making it difficult to adjust their mechanical properties and oxidation resistance simultaneously. According to the description and analysis stated above, it is reasonable to understand that macroalloying of Ti into a Mo–Si–B system can give it a better balance between the ductile phase (BCC solid solution) and the intermetallic strengthening phases (such as T_2_ and D8_8_ compounds). In the newly developed Mo–Ti–Si–B quaternary alloys, a larger volume fraction of BCC phase can be preserved even if the alloy possesses a high Si content. Moreover, the microstructure and phase equilibria can be easily tailored by varying the Ti concentration. Further optimization for the improved performance of high-temperature creep resistance, room-temperature fracture toughness and intermediate-to-high temperature oxidation resistance should be continuously focused on. The following aspects can be addressed.

1. Multi-element alloying. The BCC phase that suffers from the worst oxidation resistance should be further modified by alloying. The ductility of the alloys may also be improved through microalloying.
2. Innovation of alloy preparation process. Besides the traditional arc-melting and powder metallurgy, additive manufacturing should be tested to study the formability of this in situ compound.
3. Preparation of environmental barrier coatings. Environmental barrier coatings often act as one of the most effective methods to improve the corrosion resistance of high-temperature materials. New types of coatings should be developed for novel alloys.

In this regard, we believe that novel ultra-high-temperature structural materials beyond Ni-based or Mo–Si–B-based alloys will come to the stage in the near future.

## Appendix A

The lattice structure data of some typical compounds and their abbreviations are listed in Table A1.

**Table A1:** Crystal data for some intermetallic compounds.

Stoichiometric Formula	Abbreviations of Compound Name	Crystal Structure	Structure and Space Group
Mo_3_Si	A15	Cubic	Pm3n
Mo_5_SiB_2_	T_2_	Tetragonal	I4/mcm
Mo_5_Si_3_	T_1_	Tetragonal	I4/mcm
Ti_5_Si_3_	D8_8_	Hexagonal	I4/mcm
TiC	/	Cubic	Fm$\bar{3}$m
